# Cognitive Factors in Process Model Comprehension—A Systematic Literature Review

**DOI:** 10.3390/brainsci15050505

**Published:** 2025-05-15

**Authors:** Maximilian Möller, Michael Winter, Manfred Reichert

**Affiliations:** 1 Institute of Databases and Information Systems, University of Ulm, 89081 Ulm, Germany; manfred.reichert@uni-ulm.de; 2 Institute of Clinical Epidemiology and Biometry, University of Würzburg, 97070 Würzburg, Germany; michael.winter@uni-wuerzburg.de

**Keywords:** process model, process model comprehension, cognition, literature review

## Abstract

Process models constitute essential tools in business process management and software engineering for representing and managing real-world business processes. Hence, the proper comprehension of these models is crucial for enabling an effective and efficient communication among stakeholders. While several studies have examined factors affecting process model comprehension, such as the used modeling notation or process complexity, there is a lack of in-depth research on the cognitive processes important to comprehend process models deeper. This systematic literature review explores the cognitive mechanisms underlying process model comprehension by integrating insights from relevant disciplines such as cognitive neuroscience and psychology. Key areas of cognition include perception, attention, memory, language, problem solving, emotion, and metacognition. This review was conducted following the well-established Kitchenham methodology and included an extensive search in the following digital libraries: Web of Science, IEEE Xplore, ScienceDirect, ACM Digital Library, PubMed, and SpringerLink. By analyzing 47 studies, this literature review identifies gaps in current research, emphasizing the need for further investigation into these cognitive processes to improve model comprehensibility.

## 1. Introduction

Process models are fundamental tools in both business process management (BPM) and software engineering, serving as visual representations that help the various stakeholders (e.g., process owners, business analysts, process participants, and process/software engineers) to comprehend and manage complex processes. Both clarity and comprehensibility of these models are crucial for ensuring that it is possible to effectively and efficiently interpret and utilize the information presented by them. Studies have investigated various factors that contribute to the comprehensibility of process models, including notation, complexity, and domain knowledge. These studies have provided valuable insights into how different factors of process models affect model comprehension by humans. However, there is a significant gap in the literature concerning the cognitive processes involved in comprehending these models, particularly in how individuals mentally process, retain, and apply the information conveyed by different modeling notations and methods. There are also other critical factors that influence cognitive processing which are underexplored. For example, the layout and visual complexity of the models, the density of the information presented, and the use of colors, symbols, or hierarchical structures may have a significant impact on how humans read and comprehend the models.

Cognition, which encompasses all mental processes related to knowledge, perception, memory, and reasoning, plays a crucial role in how individuals comprehend process models. Despite the importance of process models, the specific cognitive mechanisms and processes that influence their comprehensibility are underexplored. Although the literature review by Figl [1] has explored aspects of process model comprehension, to the best of our knowledge, there is no literature review specifically focusing on the cognitive abilities involved in the comprehension of process models. Understanding these cognitive processes can provide deeper insights into how individuals interact with process models and identify ways to enhance model comprehensibility. Recent research has also explored how neural networks can support process comprehension and management—from recognizing hand-drawn BPMN models [2] to predicting service efficiency [3] and supporting business decision making [4].

The systematic literature review (SLR) presented in this article aims to highlight the areas of cognition that have already been considered in the context of process model comprehension and those that have not been thoroughly explored so far. By systematically examining existing research, this review seeks to uncover the cognitive aspects that have been addressed in the context of process model comprehension as well as to identify gaps indicating the need for further investigation. Accordingly, the following research question (RQ) is addressed in this SLR.

RQ: What cognitive factors have been examined with respect to process model comprehensibility, and where do significant research gaps persist?

The remainder of this paper is structured as follows: Theoretical backgrounds on process models, process model comprehension and cognition are presented in Section 2. Section 3 summarizes the digital libraries we queried for this review and discusses the inclusion and exclusion criteria applied to filter the relevant studies. In Section 4, the findings of this literature review are presented, followed by a discussion of the results in Section 5. Section 6 presents the threats to validity, and Section 7 concludes this paper with a summary of the key insights.

## 2. Theoretical Background

This section provides definitions and explanations for the fundamental terms used in our SLR.

### 2.1. Process Model

A process model corresponds to a formalized representation of a business process that captures the sequence of activities, events and decision points, as well as their relationships in order to achieve a specific business objective. Process models help organizations and enterprises to understand, analyze, and optimize their business processes. In essence, a process model is a structured blueprint of a business process, providing clarity on how operations unfold, on how they depend on each other, and on the conditions under which they are executed [5,6].

Process models can be classified into two main categories: declarative and imperative. Imperative models define a sequence of activities that need to be executed in the specified order. They provide a detailed prescription of how a process shall be executed. Declarative models, in turn, specify the constraints that govern a process without prescribing a specific sequence of activities. Instead of detailing the exact order of activities, declarative models allow for flexibility, as long as the defined constraints are satisfied [7,8].

**Figure 1:**
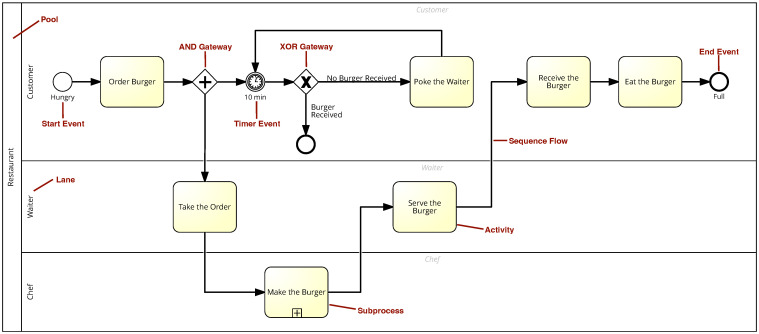
Example of a BPMN 2.0 process model.

BPMN 2.0 is a widely used standard for modeling business processes in an imperative style [9]. It uses a graphical notation to describe the flow of activities in a process, as illustrated in Figure 1.

### 2.2. Process Model Comprehension

Process model comprehension entails grasping and interpreting the information presented by a process model and thus, making sense of it. The comprehension of process models describes a cognitive process during which the reader needs to correctly understand the given concepts and identify the relevant information for the specific task [10].

Understanding how individuals comprehend process models involves delving into the cognitive processes that underpin both visual perception and interpretation. This theoretical background is built upon the principles of cognitive neuroscience and cognitive psychology, which are explained in the following.

### 2.3. Cognitive Neuroscience

In [11], cognitive neuroscience is characterized as follows,

*“Keeping up-to-date with cognitive neuroscience is much like surfing the Big Wave at Waikiki Beach. New findings keep rolling in and maintaining a stable balance is a big challenge. It is exciting, fun, and, at times, a little bit scary.”*

**Figure 2:**
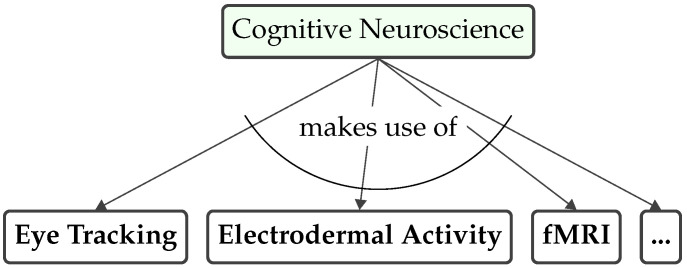
Cognitive neuroscience.

Cognitive neuroscience investigates how the brain responds to various stimuli, rapidly uncovering fresh perspectives on human cognition and the intricate workings of the mind [11]. An integral component of cognitive neuroscience is sensing technology, which encompasses measurement methods such as eye tracking or assessment of skin conductivity, also known as electrodermal activity [10]. Additional methods include Functional Magnetic Resonance Imaging (fMRI), Electroencephalography (EEG), Magnetoencephalography (MEG), Electromyography (EMG), and other techniques (see Figure 2).

The consideration of cognitive neuroscience in process model comprehension offers several benefits. First, it provides deeper insights into the cognitive processes when a viewer interacts with models, analyzing how different brain areas respond to visual and textual information. Second, advanced sensing technologies such as eye tracking and electrodermal activity measurement accurately capture cognitive load and engagement. They reveal where humans focus their attention and provide insights into their physiological arousal levels. Note that understanding how factors of process models contribute to cognitive load allows designing more comprehensible models. Cognitive neuroscience identifies aspects of a model that strain cognitive resources, informing the creation of more effective and user-friendly process models.

### 2.4. Cognitive Psychology

Cognitive psychology is the branch of science that applies the principles of epistemology to investigate how humans comprehend and react to information, including how they perceive, process, and behave in response to this information [12].

The use of cognitive psychology in comprehending (business) process models is highly advantageous. It provides a framework for comprehending the corresponding mental processes, including how humans perceive and organize visual information, which is essential for creating clear and effective designs. It further examines the limitations of human memory and information processing, guiding the creation of models that reduce cognitive strain and enhance retention. Applying these principles helps researchers and practitioners to design models that align well with natural cognitive functions, improving comprehension, usability, and effectiveness.

**Figure 3:**
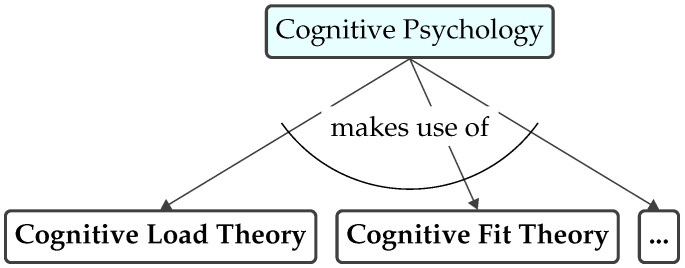
Cognitive psychology.

Cognitive psychology focuses on the practical use of methodological theories (see Figure 3) [13], of which the following five are referenced later in this article.

- Cognitive Load Theory (CLT) provides an instructional theory based on the understanding of human cognitive architecture, particularly how working memory and long-term memory interact during the learning process. CLT emphasizes the limitations of working memory, which can only hold a small amount of information at the same time over a short duration, and the virtually unlimited capacity of long-term memory, which can store vast amounts of information over extended periods.
  CLT identifies three types of cognitive load [14]:
  – Intrinsic Cognitive Load can be traced back to the characteristics of the considered content and is influenced by the complexity and interactivity of the content. The more complex and interrelated the information is, the higher is the intrinsic cognitive load.
  – Extraneous Cognitive Load is related to the way information is presented to learners. Poor instructional design might increase extraneous cognitive load by including unnecessary information or poorly structured content, which does not foster learning.
  – Germane Cognitive Load is associated with the mental effort required to create and automate mental frameworks. The construction and automation of these frameworks are crucial for transferring information from working memory to long-term memory, where it can be stored and retrieved efficiently.
- Technology Acceptance Model (TAM) was developed to explain and predict user acceptance and use of technology. TAM posits that two main factors influence an individual’s intention to use a technology: perceived usefulness (U) and perceived ease of use (EU). Perceived usefulness refers to the degree to which a person believes that using the technology will enhance its performance, whereas perceived ease of use corresponds to the degree to which a human believes that using a technology will be free of effort. Note that these factors influence the individual’s intention to use the technology, which is closely linked to its actual adoption and usage [15].
- Cognitive Theory of Multimedia Learning (CTML) presumes that people learn better with multimodel input when both visual and auditory channels are used effectively, taking into account the limited capacity of human cognitive processing. Learning becomes more effective when learners actively interact with the material by selecting, organizing, and integrating information. Key principles of CTML include using both words and pictures in combination, keeping learning resources concise, and allowing learners to control their learning pace. These guidelines foster the creation of educational materials that improve comprehension and memory retention [16].
- Cognitive Fit Theory (CFT) explains how problem solving effectiveness is influenced by the fit between task requirements and information presentation format. CFT posits that when the format of information presentation (e.g., graphs, tables) matches the cognitive processes required to solve a problem, the performance of individuals improves. This match or “fit” helps learners to efficiently utilize cognitive resources, leading to better comprehension and faster problem solving [17].
- According to Dual Coding Theory (DCT), cognition involves two distinct but interconnected systems: (1) a verbal system specialized for processing linguistic information and (2) a nonverbal system for processing imagery. The two systems operate independently as well as in orchestration to enhance learning and memory. It underscores the importance of integrating verbal and nonverbal information to foster more effective learning experiences [18].

### 2.5. Categories of Cognition

To achieve a more fine-grained understanding of cognition in the context of process model comprehension, our research divides cognition into seven foundational categories (see Table 1). These categories build upon the Cognitive Framework of Understanding and Task Performance with Diagrams (CogniDia) that was introduced by [19]. The individual categories are as follows:

- Perception. Perception is fundamental for understanding process models, as it initiates the visual processing of information and shapes the ability of the human to interpret and organize structural elements. Stimulus filtration, a critical mechanism in perception, enables humans to selectively focus on salient visual features while disregarding irrelevant details [20]. For instance, high-contrast colors and bold shapes in a process model instinctively capture attention [21], allowing viewers to identify key elements such as decision points or subprocesses. The effectiveness of perception is enhanced through Gestalt principles, such as proximity, similarity, and continuity, which help humans to intuitively group and interpret related components [22,23]. Moreover, the salience of stimuli—achieved through size differentiation, color coding, or dynamic animations—supports the prioritization of critical tasks or bottlenecks. In BPM, the alignment of visual cues with the natural flow of information facilitates the seamless navigation and comprehension, minimizing cognitive effort and reducing errors in interpretation.
- Attention and Concentration. Attention and concentration are pivotal in enabling humans to engage with process models, especially when navigating complex or intricate processes. The capacity for selective attention allows viewers to focus on relevant sections of the model while ignoring distracting elements [21]. Models that lack clarity or exhibit excessive complexity can overload cognitive resources, leading to attentional drift and comprehension failure [24]. Visual hierarchy and chunking techniques—such as grouping-related tasks, using layered structures, or highlighting critical paths—aid in directing attention to the most pertinent elements of the process [25,26]. Sustained attention, or the ability to maintain focus over time, is particularly important for analyzing intricate dependencies or interpreting lengthy sequences [27]. Additionally, clear labeling, reduced redundancy, and streamlined design contribute to maintaining human engagement [28]. In BPM, these techniques ensure that decision makers can efficiently identify dependencies, assess task priorities, and understand the overall process without unnecessary distractions.
- Memory and Knowledge Representation. This category involves the encoding, storage, and retrieval of information from process models, forming the foundation for process understanding and decision making. Working memory plays a crucial role in the immediate interpretation of process models, allowing viewers to briefly store and manipulate multiple components for analysis [24]. However, due to the limited capacity of working memory, overly detailed or poorly structured models can overwhelm it, impeding comprehension [29]. To support long-term memory integration, process models should align with the existing cognitive schemas and prior experiences of humans [30]. For example, familiar symbols, consistent visual conventions, and semantic groupings foster an easier encoding and retrieval. On the other hand, knowledge representation pertains to structuring information into mental models that are both coherent and actionable. Effective representations leverage hierarchical structures or network structures, enabling humans to conceptualize relationships and dependencies. In BPM, clear and well-structured models enhance recall, support reasoning, and promote the transfer of knowledge to new or evolving scenarios.
- Language. Language processing is integral to understanding process models, as it bridges the gap between visual and textual elements. Clarity, brevity, and precision in textual process descriptions, task labels that describe specific process steps, and model annotations that provide additional explanations about process elements significantly improve comprehension. Ambiguity in language—such as vague task labels or inconsistent terminology—can disrupt process model understanding and lead to misinterpretation of process flows. Semantic alignment between text and visual elements ensures coherence, with textual descriptions directly corresponding to their visual counterparts [31]. Additionally, the use of domain-specific terminology tailored to the target audience enhances relevance and model comprehension. For example, employing industry-specific language in process models for a specialized audience fosters a deeper engagement and facilitates decision making. Incorporating instructional elements, such as contextual explanations or tooltips, further supports model comprehension by reducing cognitive load and helping humans connect the textual information with their overall understanding of how the process works.
- Problem Solving and Decision Making. In the context of process models, problem solving and decision making involve the application of cognitive strategies to analyze, evaluate, and address specific challenges within a process, such as identifying bottlenecks, resolving inefficiencies, managing resource dependencies, and ensuring process compliance [32]. The identification of bottlenecks, inefficiencies, or dependencies requires humans to systematically decompose the model into manageable components. Critical thinking and analytical reasoning are essential for evaluating alternative processes, optimizing processes, and predicting outcomes such as improved efficiency, reduced costs, and enhanced process reliability. Process models that visually highlight dependencies—such as showing clear connections between tasks, indicating conditional paths for different scenarios, or organizing tasks in a clear hierarchy—make it easier for humans to identify potential conflicts or inefficiencies within the process. Additionally, simulating models and analyzing different scenarios can support decision making by allowing humans to test hypothetical modifications and assess their impact on the process. Effective process models act as cognitive aids, guiding humans through structured reasoning and enabling them to make informed, data-driven decisions.
- Emotion and Motivation. Emotional and motivational factors play a significant role in how humans interact with process models and how they comprehend them. Positive emotional states, such as curiosity or a sense of accomplishment, enhance cognitive flexibility and engagement, fostering deeper exploration and understanding [33]. Conversely, negative emotions, such as frustration or anxiety, can hinder comprehension and lead to disengagement, particularly when models are overly complex or poorly designed [34]. Motivation also plays a key role, providing the drive and persistence needed to carefully navigate and analyze complex models. Intrinsic motivation, such as the desire to solve a problem or achieve a goal, can be supported by intuitive and visually appealing model designs. Providing positive reinforcement, such as clear feedback or visual cues like progress bars and step indicators, can further enhance motivation and emotional engagement. In BPM, designing models that balance clarity and challenge helps maintain human interest and fosters a productive interaction with the process [35].
- Metacognition and Self-regulation. Metacognition and self-regulation involve the monitoring and control of the cognitive and emotional states of a human during the comprehension of process models. Metacognitive strategies involve planning how to approach the interpretation of process models, assessing the level of understanding, and identifying potential difficulties in comprehension [36]. For example, humans might employ chunking techniques to break down complex models into more manageable subcomponents. Self-regulation complements these strategies by managing emotional responses, such as frustration, and by keeping focus on the task at hand [37]. Process models can support metacognition by providing contextual aids, such as visual summaries, hierarchical views, or interactive elements that allow humans to explore model details at their own pace. Additionally, scaffolding strategies, such as guiding questions or progressive disclosure of information, can assist humans in navigating through complex processes. In BPM, fostering metacognitive skills is crucial for enabling humans to interact and adapt with models and overcome challenges, ultimately leading to more effective decision making and problem solving [38].

This section has outlined the comprehension of process models and the cognitive mechanisms involved. To further explore these aspects, a structured and methodical approach becomes essential. Section 3 provides a description of the methodology, ensuring a systematic and reproducible procedure for identifying the pertinent literature in the field of process model comprehension and cognition.

## 3. Research Method

This section summarizes the methodology of the SLR, the digital libraries considered, and the SLR search string, as well as the inclusion and exclusion criteria applied to filter relevant studies.

### 3.1. Methodology

A systematic and structured approach was employed to select and source the literature, ensuring a comprehensive review that addresses both the scope and depth of the research topic. The SLR was conducted according to the rigorous protocol established by Kitchenham et al. [39], which is widely recognized in the fields of software engineering and information systems. This protocol outlines a three-phase methodology, as described below.

Phase 1: Planning

The planning phase involves developing a solid review protocol. Key tasks are to define the objectives of the SLR, to formulate a precise query for searching databases, to select relevant digital libraries for retrieving studies, and to carefully establish inclusion and exclusion criteria to filter the gathered research effectively.

Phase 2: Conducting

This phase executes the search strategy and evaluates the retrieved literature. The process starts with applying the developed query across the selected digital repositories, yielding a collection of academic articles. Each article is then rigorously reviewed by three experts in information systems, applying pre-defined inclusion and exclusion criteria to ensure a precise and relevant selection. To expand the dataset, backward and forward snowballing techniques are employed [40]—backward snowballing reviews the references of selected studies, whereas forward snowballing identifies studies that cite them. Both methods follow the same strict criteria. Finally, data are extracted from the selected articles, categorized, and thematically grouped to address the predefined research questions.

Phase 3: Reporting

The final phase documents and presents the findings of the review. This includes synthesizing and articulating the results in a clear and structured manner, with a focus on answering the research questions that were formulated at the outset.

### 3.2. Digital Libraries and Search String

The systematic literature search was conducted in May 2024 and queried six digital libraries: Web of Science, IEEE Xplore Digital Library, ScienceDirect, ACM Digital Library, PubMed, and SpringerLink. The following search criteria were used:

- Search fields: Metadata (title, abstract, key words).
- Search string (The * character was used to search for variations of terms, for example, not only for the term “cognitive” but also for “cognition”): (“process model*”) AND (understand* OR comprehen*) AND (cogniti*).
- Timespan = none.

**Figure 4:**
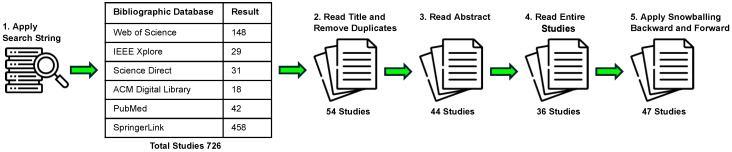
Collection of studies.

The search string was modified according to the library, as it was only feasible to limit the search field in certain libraries. The studies collection process is shown in Figure 4.

### 3.3. Inclusion and Exclusion Criteria

All studies that explicitly deal with the cognitive processes involved in reading and comprehending process models were included. It did not matter whether the cognitive processes were investigated with the help of an empirical study or theoretically. By incorporating both empirical and theoretical studies, the SLR provides a comprehensive synthesis of diverse insights. Empirical studies provide concrete evidence and practical insights, whereas theoretical studies offer foundational frameworks and hypotheses that can help when interpreting empirical findings.

Following the literature review conducted by Figl [1], studies are excluded if they do not focus on procedural (i.e., imperative) process models. Procedural process models describe processes through a defined sequence of steps, specifying the exact order of activities required to achieve a particular outcome. Well-known examples include BPMN 2.0 and flowcharts, which provide a clear and structured representation of processes. Consequently, all studies dealing with conceptual (process) models other than imperative ones were excluded. These models have different structures and underlying principles, which could introduce variability in process representation and confound the analysis of cognitive processes specific to procedural models.

All papers dealing with cognitive aspects of process model creation (i.e., the process of process modeling) were excluded, i.e., this SLR focuses on the cognitive process of process model comprehension but not of process model creation. This clear focus allows drawing more precise conclusions about the cognitive mechanisms applied during the reading and comprehending phase.

### 3.4. Resulting Studies

Applying the search string to the above digital libraries resulted in a total of 726 studies. A manual scan for relevance by reviewing the titles reduced the number of studies to 54. After reading the abstracts, 44 studies remained. Each of these studies was then entirely read, whereby further studies could be excluded, resulting in 36 relevant studies. According to [40], snowballing was applied. In the first iteration of snowballing, 11 further studies were identified (2 backward and 9 forward). As no new studies were found in the second iteration, snowballing was terminated. Finally, 47 studies resulted (cf. Table A1, Table A2, Table A3, Table A4, Table A5, Table A6 and Table A7) serving as basis of this SLR. By applying the above methodology, a comprehensive collection of studies that delve into the cognitive processes underlying process model comprehension has been collected.

## 4. Reporting the Results

This section provides an overview of the research clusters identified from the collected studies and presents the results of the SLR. The focus lies on the cognitive factors that affect process model comprehension, empirical studies utilizing methodologies from cognitive neuroscience and psychology, and the modeling notations used.

### 4.1. Research Clusters

**Figure 5:**
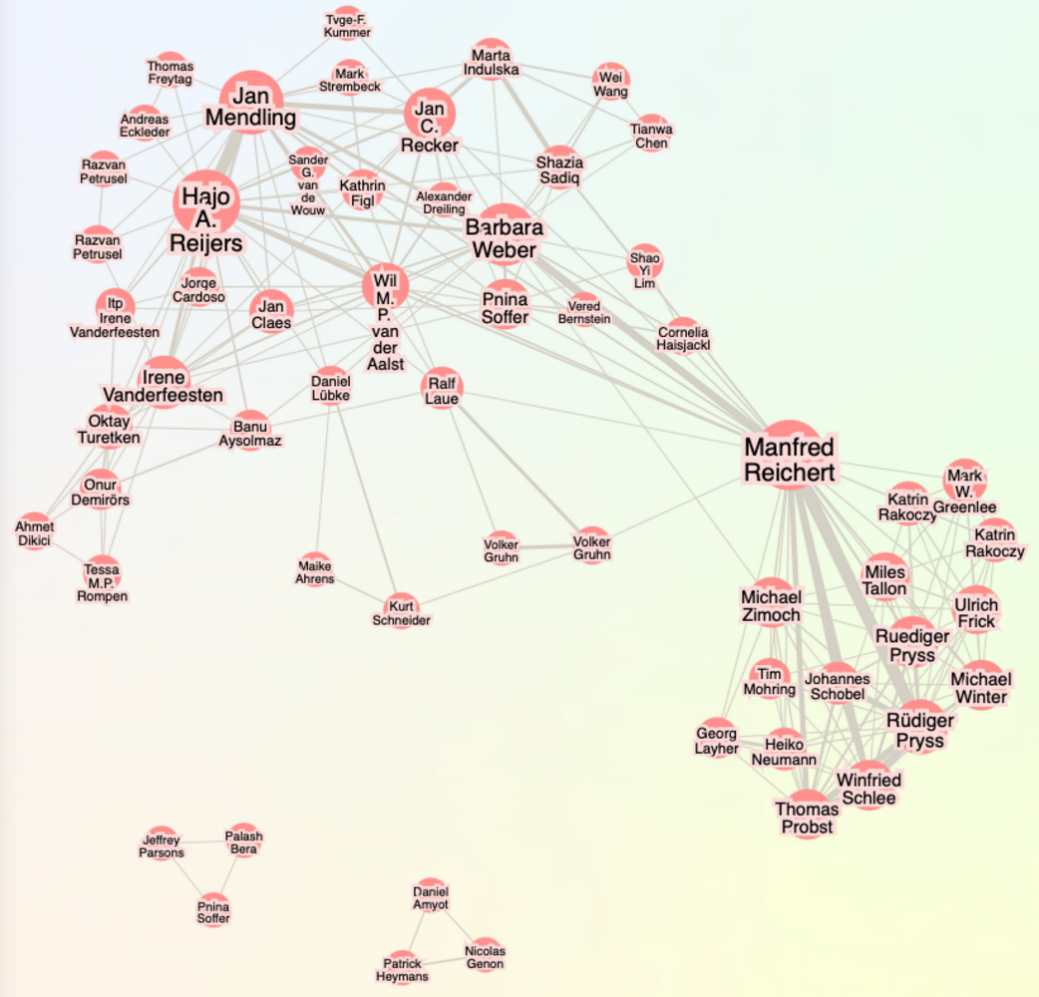
Identified research clusters.

A closer examination of the authorship and collaborations reveals a clustering of researchers into distinct groups, reflecting the collaborative nature of academic endeavors. The network of researchers, as depicted in Figure 5, can be categorized into three main clusters.

- Research cluster I. The first research cluster, led by Manfred Reichert, demonstrates a particularly dense network with strong collaborations involving researchers such as Rüdiger Pryss, Michael Winter, and Ulrich Frick.
- Research cluster II. The second research cluster is centered around Jan Mendling, Hajo A. Reijers, and Barbara Weber, who are closely interconnected through significant co-authorship ties. This cluster also includes notable collaborators such as Wil M.P. van der Aalst, Jan C. Recker, and Irene Vanderfeesten.
- Research cluster III. The third research cluster consists of less researchers who are not as strongly interconnected as clusters I and II. These include, for example, the cluster of Jeffrey Parsons, Palash Bera, and Pnina Soffer, as well as the cluster formed by Patrick Heymans, Nicolas Genon, and Daniel Amyot. These researchers represent more independent collaborations within the field.

Table 2 assigns the considered studies to the identified research clusters.

### 4.2. Cognitive Factors in Process Model Comprehension

We assign the considered studies to the categories of cognition set out in Section 2. This categorization is presented in Table 3.

Perception is addressed by almost all studies, indicating its fundamental importance in cognitive research. Many studies examine how the layout and visual design of process models affect model comprehension (e.g., S12, S29, S34, and S37). Model elements such as the sequencing of activities or the use of colors, shapes, and symbols are crucial for supporting humans to easily perceive and comprehend the model. An appropriate layout design can reduce cognitive load and help humans to quickly grasp complex information. The complexity of process models, often measured in terms of the number of activities, branches (i.e., splits and joins in the flow of activities), and interconnections (i.e., the connections between process elements such as activities and events), significantly impacts how humans perceive process information. Research on process model comprehension often focuses on how different levels of complexity influence the ease with which humans can understand and use the models (e.g., S6, S8, S12, and S18). Simplifying complex models or finding ways to visually represent them more appropriately is a common research goal (see Table 3, Column 2).

Attention and Concentration are featured prominently in about half of the studies, reflecting significant interest in how focus and sustained attention impact cognitive tasks. One explanation for this is that several studies used eye-tracking technology to measure the attention of participants (e.g., S1–S4, S6, and S9). Eye tracking provides precise data on where and how long humans focus their gaze on specific elements of a process model, allowing researchers to gain valuable insights into attentional patterns and cognitive engagement during the interpretation of these process models. However, attention was not only measured in terms of eye tracking. Other aspects such as perceptual discriminability and semantic transparency were also employed to assess how well humans could focus on the information presented in process models and comprehend it (e.g., S17 and S21). Perceptual discriminability enables that humans can easily differentiate between various elements of the model, reducing confusion and improving comprehensibility. Semantic transparency ensures that the symbols and icons used in the models intuitively convey their meaning, facilitating quicker comprehension (see Table 3, Column 3).

Memory and Knowledge Representation are widely covered, underscoring their critical role in understanding how information is stored and retrieved. Most studies in this field have compared novices and experts to explore how different levels of experience influence cognitive processing and comprehending of process models (e.g., S1–S4 and S44). Note that not all studies considered the differences between novices and experts but investigated memory and knowledge representation in other ways. For example, some studies use modularization (i.e., breaking down complex information into manageable, independent modules) to reduce cognitive load and enhance memory retention (e.g., S9 and S15), while others (e.g., S12) examined gaze patterns and visual routines to understand how visual information is processed and retained. Research also explored the role of working memory, visual attention distribution, and modularization strategies in the comprehension of process models (e.g., S14 and S16). Additionally, frameworks were developed (e.g., S13) to measure how process models were understood by different stakeholders, emphasizing the role of prior knowledge and cognitive schemas (see Table 3, Column 4).

Language is less frequently discussed. Only a few studies explored various aspects of linguistic process model elements. For example, they have investigated the impact of abstract versus concrete labels on comprehending process models (e.g., S25). Furthermore, researchers examined the integration of linguistic elements such as narration or verbal explanations to enhance comprehension by reducing cognitive load and improving information processing through dual coding theory (e.g., S38). The influence of cultural differences in the interpretation of linguistic elements was also a focus (e.g., S29), demonstrating how these factors affect cognitive processing and understanding (see Table 3, Column 5).

Problem Solving and Decision Making are emphasized by numerous studies, indicating their importance in cognitive research related to analytical and decision-making processes. This category is common, as in many studies (e.g., S6–S7 and S9–S12), participants were asked to answer questions about the process models, which naturally involves problem solving. For instance, participants were asked to determine the next steps in a process, to identify potential bottlenecks, or to assess the efficiency of a process. These tasks require participants to engage in problem-solving activities, e.g.,:

- analyzing the process model
- making decisions
- identifying problems

The inclusion of such comprehension questions ensures that participants actively engage with the process models, applying their cognitive skills to solve problems as well as to make decisions. This approach not only tests their comprehension but also provides insights into how well the process models support effective problem solving and decision making (see Table 3, Column 6).

Emotion and Motivation are addressed in fewer studies, indicating that these factors are considered less often but are still recognized for their impact on cognition. Some studies have examined the use of serious games to motivate participants and enhance their model comprehension (e.g., S7). The measurement of electrodermal activity to understand cognitive load and arousal levels during model comprehension were investigated in other studies (e.g., S8). These studies reveal how complex models increase electrodermal activity, reflecting higher cognitive load and emotional states like stress and attention. Research also utilized eye-tracking and think-aloud methods (e.g., S16) to capture participants’ cognitive and emotional strategies in real time, highlighting their motivational states during model comprehension (see Table 3, Column 7).

Metacognition and Self-regulation are covered by a smaller number of studies, indicating a growing interest in how individuals monitor and control their cognitive processes. For example, studies (e.g., S16) have shown that participants verbalized their thoughts, actively being engaged in monitoring their comprehension, and adjusting their strategies as needed. This real-time feedback and strategy adjustment reflects self-regulation and metacognition. Participants adapted their inspection strategies to improve accuracy and efficiency, demonstrating active regulation of their cognitive processes. Additionally, by tracking eye movements and analyzing verbalizations, research revealed how participants monitored and controlled their cognitive processes, assessed their comprehension, and adapted their strategies to effectively comprehend process models (see Table 3, Column 8).

### 4.3. Empirical Studies

This subsection deals with the methodologies from cognitive neuroscience and cognitive psychology examined in the studies.

Methodologies from Cognitive Neuroscience: Sensor technologies from cognitive neuroscience were used by 19 studies (i.e., 46.3% of the empirical studies). Out of the 19 studies, 18 of them used eye tracking and one employed the method of electrodermal activity. None of the selected studies relied on advanced neuroimaging and electrophysiological methods like fMRI, EEG, MEG, or EMG.

Eye Tracking. Eye tracking is a technique used to study visual attention by tracking where and for how long a person looks at specific objects. It also records the scan path, showing the sequence of eye movements. This method is applied in fields such as cognitive psychology, marketing, and human–computer interaction (including human factors). In user experience research, eye tracking helps gain insights into user interactions that cannot be captured through verbal descriptions alone [85].

In the literature review [86], which focuses on the use of eye tracking, four metrics were considered: fixations, saccades, scan path, and duration. These metrics, except for duration, were identified and considered in our SLR (see Table 4) as well. Duration was not considered, as it does not necessarily constitute a metric associated with eye tracking. This aligns with the findings reported by [87], which describe these three metrics as the most frequently ones used in the context of eye tracking.

In all studies that used eye tracking, fixations were measured, either as the number of fixations or the fixation duration. A fixation happens when the eye stops moving and focuses on a specific spot in the visual field [85]. In five studies, in addition to the measurement of fixations, saccades were examined. Saccades constitute rapid shifts of the eye from one point to another, which help when piecing together a complete image of the viewed scene [85]. Emphasized by Study S1 [13], it is important to understand that during saccadic eye movements, the eye does not capture any visual input. In nearly half of all studies that used eye tracking, the scan path was applied as a metric. A scan path (or gaze path) shows the route the eyes follow, including the sequence of fixations and saccades. This scan path can reveal how visual information is processed.

Electrodermal activity. Study S8 [47] explored whether electrodermal activity can be used to assess cognitive load during process model comprehension. A preliminary test run is performed as well as a feasibility study with nine participants who had to understand two different BPMN 2.0 process models while recording their electrodermal activity. S8 measured the mean skin conductance level (SCL) to assess variations in physiological arousal during the comprehension of process models, indicating whether different levels of complexity lead to increased cognitive load. The number of skin conductance response (SCR) peaks was analyzed to identify moments of high physiological or psychological demand, evaluating whether more peaks occurred with increasing complexity of the models. Additionally, the mean of SCR amplitudes energy level was measured to determine the level of stress caused by the task of comprehending process models. In this context, higher amplitudes signify greater cognitive load.

Methodologies from Cognitive Psychology. Out of the 41 empirical studies, 37 use (methodological) theories from cognitive psychology. Table 5 shows the distribution of the theories across the studies. In all studies, CLT (see Section 2.4) was used. The second most frequently used theory was CTML (S22, S24, S38, S44, S45, S47), followed by TAM (S7, S8, S11, S12, S15, S16). Finally, CFT was utilized by the empirical studies (S24, S26, S40, S45, S47), as well as DCT (S38, S45).

Notation. The empirical studies were analyzed based on the notation used. The notations were split into three categories: BPMN, Non-BPMN, and Both. Note that BPMN 2.0 is a de facto industry standard (ISO/IEC 19510:2013 [88] norm) for creating and visualizing process models, which is broadly used in both industry and academics. The first category included all studies that use BPMN (including its current version, BPMN 2.0). The second category, Non-BPMN, comprised all studies that did not employ BPMN but instead examined other notations such as Event-driven Process Chains (EPC), Yet Another Workflow Language (YAWL), and Unified Modeling Language activity diagrams (UML AD). These notations were explicitly analyzed to allow for a comparative perspective on the cognitive effects across different modeling languages. The last category included studies that examined both BPMN and other notations.

Table 6 shows the distribution of the studies by notation. The majority of empirical studies consider BPMN (i.e., 70.7% of the empirical studies analyzed). A small portion of the research, comprising four studies, used notations other than BPMN. This accounts for 9.8% of the total studies. There are eight studies that employed both BPMN and other notations, making up 19.5% of the empirical studies analyzed.

### 4.4. Theoretical Discussion

This subsection deals with studies that do not involve an empirical study but deal with cognition in the area of process model comprehension from a theoretical perspective.

S19 [58] deals with the application of cognitive complexity measures to process models. The study adapts a cognitive complexity measure originally developed for software code, proposing its use for evaluating the comprehensibility and maintainability of process models. The study presents cognitive weights for various process model elements, i.e., sequence, splits, and joins, using workflow nets/YAWL as a modeling language. S19 aims to provide a more nuanced measure of process model complexity that accounts for human cognitive processing.

S20 [59] presents a theoretical framework that divides the learning process into three stages: Presage (user characteristics), Process (knowledge construction), and Product (learning outcome). S20 emphasizes that comprehending process models is not only influenced by the models themselves but also by characteristics of humans such as motivation, skills, and experience. The framework aims to enhance model comprehension by considering cognitive psychology, goal-setting theory, and multimedia learning theory. It also provides recommendations for improving both process models and user training. This integrative approach sought to bridge the gap between model design and user interaction, fostering more effective use of process models in practice.

S25 [19] explores how cognitive factors influence the comprehension and task performance in comprehending diagrams in systems analysis and design. S25 consolidates cognitive research to develop a theoretical framework, called CogniDia, which outlines the cognitive processes involved in model comprehension and task performance with diagrams. The CogniDia framework integrates various cognitive theories from software and information systems engineering to classify and review the criteria needed for effective diagram comprehension and task performance.

S13 [52] aims to address the challenge of measuring and quantifying how well process models are understood by humans. S13 introduces the Process Model Comprehension Framework (PMCF), which includes 96 quality metrics to quantify process model comprehension. PMCF serves as a tool to identify and address issues in process model comprehension, ultimately aiming to enhance the effective use of process models in practice.

S21 [60] evaluates the cognitive effectiveness of BPMN 2.0 using principles from the Physics of Notations theory. S21 systematically analyzes BPMN 2.0 against nine principles of an effective notation (e.g., cognitive fit, perceptual discriminability, cognitive integration, or visual expressiveness) to identify strengths and weaknesses of the BPMN 2.0 notation.

S23 [62] investigates the influence of hierarchy on the comprehensibility of conceptual models. S23 reviewed existing empirical studies, which show mixed results regarding the benefits of modularization. S23 proposes a framework based on cognitive psychology, highlighting two opposing forces: abstraction, which reduces mental effort, and the split-attention effect, which increases mental effort by requiring context switches between sub-models. Its framework aims to explain the conditions under which hierarchy improves or impairs comprehensibility.

Overall, these theoretical studies contribute valuable insights into the cognitive processes involved in process model comprehension by offering frameworks, cognitive measures, and theoretical models.

## 5. Discussion and Future Work

This section answers the research question introduced in Section 1, including the discussion of the research gaps, the implications for researchers and practitioners, and future work.

### 5.1. Answering the Research Question

The findings of our SLR highlight the crucial role cognitive processes play in the comprehension of process models. Therefore, our research question is answered by several cognitive factors that have been extensively examined, such as Perception, which focuses on how visual elements like colors, shapes, and layouts are perceived and interpreted.

RQ: What cognitive factors have been examined with respect to process model comprehensibility, and where do significant research gaps persist?

Attention and Concentration have been studied through eye-tracking methods, providing valuable insights into patterns of the attention and cognitive engagement of the humans.

Research on Memory and Knowledge Representation has, in parallel, highlighted the importance of working memory and cognitive schemas in processing and retaining information from process models.

Problem Solving and Decision Making have been highlighted through empirical studies, which frequently include problem-solving tasks to evaluate comprehension, showcasing the analytical processes involved.

While some cognitive factors have been thoroughly investigated, others remain comparatively underexplored. Only limited studies exist that investigate the impact of Language in process models, e.g., the role of text annotations and labels of process elements in enhancing comprehension.

Moreover, Emotion and Motivation are acknowledged; however, their role in process model comprehension remains underexplored, even though they have the potential to affect cognitive engagement, sustain attention, and influence the depth of processing, all of which are critical for effectively reading and understanding process models.

Only a few studies have investigated Metacognition and Self-regulation, e.g., how individuals monitor and control their cognitive processes when interacting with process models—yet this is a crucial area, as metacognitive abilities enable learners to plan, monitor, and evaluate their comprehension strategies, helping them to detect misunderstandings, adjust their focus, and allocate mental resources more effectively, which in turn can significantly improve the accuracy and depth of process model understanding. Our findings significantly contribute to the broader field of process model comprehension by highlighting the multifaceted nature of the cognitive processes involved. The detailed exploration of the perception, attention, and memory offers a deeper understanding of how visual and cognitive elements interact to influence comprehension. By identifying underexplored areas, this review sets the roadmap for future research to adopt a more holistic approach to the comprehension of cognitive processes when reading process models. Similarly, understanding emotional and motivational factors provides insights into how they influence cognitive engagement, potentially leading to the development of more engaging and user-friendly process models.

### 5.2. Implications

The implications of the presented results are relevant for both researchers and practitioners.

#### 5.2.1. Theoretical Implications

There is a need to broaden the scope of studies to investigate underexplored cognitive categories such as language, emotion, and metacognition. Integrating advanced sensing technologies like functional Magnetic Resonance Imaging (fMRI) or Electroencephalography (EEG), alongside traditional methods like eye tracking, will enable a more complete picture of cognitive processes. FMRI provides insights into neural activation patterns, allowing researchers to investigate how different regions of the brain are engaged during process model comprehension, particularly in relation to complex cognitive functions like attention and working memory. In turn, EEG offers a high temporal resolution, meaning it can measure brain activity with millisecond precision, allowing researchers to track rapid cognitive processes and identifying changes in brain activity in real time.

Although these sensing technologies hold significant potential for uncovering the neural and cognitive mechanisms underlying process model comprehension, they have remained underrepresented in current research. Specifically, in this review, fMRI and EEG have not been included as methodological approaches, primarily due to the lack of existing studies leveraging these technologies. Involving fMRI and EEG in future studies could provide a more precise understanding of how cognitive load, emotional responses, and individual differences influence the human engagement in process model comprehension. These insights could lead to the development of adaptive strategies and tools that address diverse cognitive needs, enhancing both theoretical understanding and practical applications in the field of process model comprehension. Additionally, investigating individual differences in cognitive styles and preferences can contribute to the development of tailored strategies that enhance process model comprehension across various stakeholders.

#### 5.2.2. Practical Implications

The insights from cognitive research gained in this review can directly influence the design of process models in several ways. By incorporating findings on perception, practitioners can optimize the visual layout of process models. For example, using clear visual representations, consistent color schemes, and grouping-related elements can reduce cognitive load and enhance the ability of humans to navigate and comprehend complex models. Eye-tracking studies, as discussed in this review, suggest that using visual cues (e.g., bold outlines or highlighting) to emphasize critical paths or key decision points in process models can effectively guide human attention and enhance task performance.

Understanding cognitive factors, as discussed in Section 2, helps in developing training programs tailored to the way humans process and retain information. Training materials can incorporate strategies such as chunking information, utilizing dual coding (for combining i.e., text and images), and leveraging interactive modules that allow humans to explore process models at their own pace. These approaches align with cognitive load theory and reflect the key findings of this review, which emphasize the importance of reducing cognitive strain for better knowledge retention and practical application.

Moreover, findings on emotional and motivational factors provide actionable insights for creating more engaging process models. For instance, integrating elements such as progress indicators, feedback mechanisms, and gamification techniques enhance human motivation and maintain interest, leading to a more profound process model comprehension. Addressing emotional barriers such as frustration or anxiety through intuitive designs, as highlighted in this review, can improve stakeholder satisfaction and collaboration as well.

These practical insights from cognitive research on process model comprehension, as derived from the findings of this review, empower practitioners to design process models not only being effective in conveying information but also being adaptable to diverse human needs, fostering greater understanding, usability, and stakeholder involvement.

### 5.3. Future Work

Future research should focus on the underexplored cognitive factors, such as language, emotion, and metacognition, to provide a more holistic understanding of the cognitive processes that are involved in process model comprehension.

Expanding the use of sensing technologies is also important. While eye tracking is a well-established technology, integrating advanced technologies such as fMRI, EEG, and physiological measures can provide deeper insights into brain activities and cognitive load. Combining different sensing technologies can provide a comprehensive view on cognitive processes. For example, integrating eye tracking with fMRI or EEG can correlate the visual attention with the brain activity, enabling more profound insights into cognitive mechanisms.

Additional research is needed in the intraindividual direction, exploring how individual differences affect process model comprehension and the strategies that can be employed to accommodate these differences. Individual differences, such as cognitive style, prior knowledge, and personal experience, play a significant role in how process models are comprehended. For instance, some individuals may have a preference for visual information, while others can better comprehend textual descriptions. According to CLT, these differences influence how information is processed and retained.

This personalized approach can enhance comprehension by reducing cognitive load and improving information retention.

## 6. Threats to Validity

Despite providing valuable insights into the cognitive processes involved in process model comprehension, several threats to validity must be considered. This review primarily focuses on selected cognitive categories, which may have led to the exclusion of other relevant cognitive factors. The concentration on areas such as perception, attention, and memory might have overshadowed other important aspects like creativity and intuition. The present literature review was conducted using a specific set of digital libraries (see Section 3). This approach might have resulted in the exclusion of relevant studies indexed in other libraries. Additionally, the used search string was specified to metadata fields such as title, abstract, and keywords. This might have led to the omission of studies where relevant information was not prominently featured in these fields but was nonetheless crucial to understanding cognitive processes in process model comprehension. Using different search strings or broader criteria could have resulted in the identification of other relevant studies. To reduce selection bias, we conducted a structured, multi-stage screening procedure involving three researchers with relevant domain expertise. The entire retrieval process—including title, abstract, and full-text screening—was performed independently by all three researchers. Disagreements were resolved through group discussion and mutual consensus. Although formal inter-rater reliability (e.g., Cohen’s Kappa) was not calculated, this collaborative and multi-analyst procedure was intended to ensure consistency, reduce subjectivity, and enhance the robustness of the study selection. Finally, while we analyzed cognitive processes individually to allow for clearer categorization and comparison across studies, we acknowledge that this approach does not fully reflect the dynamic interplay between these processes in real-world cognitive tasks. We highlight the importance of investigating cognitive integration and interaction in future research on process model comprehension.

## 7. Conclusions

Overall, this SLR has laid the groundwork for a more comprehensive and detailed exploration of cognitive processes during process model comprehension. By addressing the identified gaps and limitations, future research can contribute to the development of more effective and human-centered process models, ultimately improving stakeholder engagement, decision making, and the practical application of process models in business and software engineering contexts. Enhanced understanding of these cognitive processes will not only inform the design of process models but also lead to better training and support for humans, facilitating more efficient and effective utilization of process models in real-world scenarios. Furthermore, the integration of findings from cognitive science into the design and implementation of process models holds the potential to significantly advance the field, driving innovation and improving outcomes across various domains where process models are applied. As technology and methodologies evolve, continuous research will be essential to maintain the relevance and efficacy of process models.

## Figures and Tables

**Table 1:** Seven categories of cognition.

Category	Description
Perception	How are visual features like colors, shapes, and layout in process models perceived to enable best possible comprehension.
Attention and Concentration	Vital for focusing on relevant aspects of process models, with factors such as model complexity and visual clarity influencing focus and requiring consistent attention to understand intricate details.
Memory and Knowledge Representation	How is information from process models encoded, stored, and retrieved, and how do cognitive schemas and prior experience influence the way this information is integrated into mental models for reasoning and decision making.
Language	Involves understanding textual descriptions and labels in process models, where clear language enhances interpretation of visual elements and overall comprehension.
Problem Solving and Decision Making	Involve analyzing task dependencies, identifying issues, and using cognitive processes to evaluate alternatives and select optimal solutions.
Emotion and Motivation	Influence process model comprehension, with positive emotions and high motivation enhancing engagement, while negative emotions may impede comprehension.
Metacognition and Self-regulation	Involve monitoring comprehension, adjusting strategies, and managing focus and motivation to effectively comprehend process models.

**Table 2:** Research clusters and studies identified in the SLR.

Research Cluster	Studies	# of Studies
I	S1–S16	16
II	S17–S20, S22–S26, S28–S46	28
III	S21, S27, S47	3

**Table 3:** Studies distribution by cognitive processes.

Study	Source	Perc.	Att. and Conc.	Mem. and Know. Rep.	Lang.	P.S. and D.M.	Emot. and Motiv.	Meta. and Self-Reg.
S1	[13]	✓	✓	✓		✓		
S2	[41]	✓	✓	✓				
S3	[42]	✓	✓	✓		✓		
S4	[43]	✓	✓	✓				
S5	[44]	✓		✓				
S6	[45]	✓	✓	✓	✓	✓		
S7	[46]	✓		✓		✓	✓	
S8	[47]	✓	✓			✓	✓	
S9	[48]	✓	✓	✓		✓		
S10	[49]	✓	✓			✓		
S11	[50]		✓	✓		✓		
S12	[51]	✓	✓	✓		✓		
S13	[52]	✓		✓				
S14	[53]		✓	✓		✓		
S15	[54]	✓	✓	✓		✓		
S16	[55]	✓	✓	✓		✓	✓	✓
S17	[56]	✓	✓	✓		✓		
S18	[57]	✓		✓		✓		
S19	[58]	✓						
S20	[59]	✓		✓		✓	✓	
S21	[60]	✓	✓	✓				
S22	[61]	✓	✓	✓		✓		
S23	[62]	✓	✓	✓		✓		
S24	[63]	✓	✓	✓		✓		
S25	[19]	✓	✓	✓	✓	✓	✓	
S26	[64]			✓				
S27	[65]	✓	✓	✓		✓		
S28	[66]	✓				✓		
S29	[67]	✓	✓	✓	✓	✓		
S30	[68]		✓			✓		
S31	[69]		✓	✓	✓	✓		
S32	[70]	✓	✓	✓	✓	✓		
S33	[71]	✓	✓	✓	✓	✓		✓
S34	[72]	✓			✓			
S35	[73]	✓	✓	✓		✓		
S36	[74]	✓		✓		✓		
S37	[75]	✓	✓			✓		
S38	[76]	✓	✓		✓	✓		
S39	[77]	✓						
S40	[78]	✓	✓	✓	✓	✓		
S41	[29]	✓		✓		✓		
S42	[79]	✓						
S43	[80]	✓		✓		✓		
S44	[81]	✓		✓		✓		
S45	[82]	✓	✓	✓		✓		
S46	[83]	✓	✓			✓		
S47	[84]	✓	✓			✓		✓

**Table 4:** Eye-tracking metrics used by studies.

Study	Source	Fixations	Saccades	Scan Path
S1	[13]	✓		✓
S2	[41]	✓	✓	✓
S3	[42]	✓	✓	✓
S4	[43]	✓	✓	✓
S6	[45]	✓		
S9	[48]	✓		
S10	[49]	✓		✓
S11	[50]	✓		
S12	[51]	✓	✓	✓
S15	[54]	✓		
S16	[55]	✓		
S24	[63]	✓		
S27	[65]	✓		
S30	[68]	✓	✓	
S35	[73]	✓		✓
S37	[75]	✓		
S40	[78]	✓		✓
S47	[84]	✓		

**Table 5:** Methodological theories.

Study	Source	CLT	TAM	CTML	CFT	DCT
S1	[13]	✓				
S2	[41]	✓				
S3	[42]	✓				
S4	[43]	✓				
S5	[44]	✓				
S6	[45]	✓				
S7	[46]	✓	✓			
S8	[47]	✓	✓			
S9	[48]	✓				
S10	[49]	✓				
S11	[50]	✓	✓			
S12	[51]	✓	✓			
S15	[54]	✓	✓			
S16	[55]	✓	✓			
S17	[56]	✓				
S22	[61]	✓		✓		
S24	[63]	✓		✓	✓	
S26	[64]	✓			✓	
S27	[65]	✓				
S28	[66]	✓				
S45	[82]	✓		✓	✓	✓
S46	[83]	✓				
S29	[67]	✓				
S31	[69]	✓				
S32	[70]	✓				
S33	[71]	✓				
S34	[72]	✓				
S35	[73]	✓				
S36	[74]	✓				
S37	[75]	✓				
S38	[76]	✓		✓		✓
S40	[78]	✓			✓	
S42	[79]	✓				
S44	[81]	✓		✓		
S47	[84]	✓		✓	✓	

**Table 6:** Studies distribution by notation.

Notation	Source	# Studies (%)
BPMN	S1 [13], S4 [43], S6 [45], S7 [46], S8 [47], S9 [48], S10 [49], S11 [50], S12 [51], S14 [53], S15 [54], S16 [55], S22 [61], S24 [63], S26 [64], S27 [65], S28 [66], S29 [67], S30 [68], S32 [70], S33 [71], S34 [72], S35 [73], S36 [74], S37 [75], S38 [76], S40 [78], S46 [83], S41 [29]	29 (70.7)
Non-BPMN	S3 [42], S18 [57], S31 [69], S43 [80]	4 (9.8)
Both	S2 [41], S5 [44], S17 [56], S39 [77], S42 [79], S44 [81], S45 [82], S47 [84]	8 (19.5)

## Abbreviations

The following abbreviations are used in this manuscript:

BPM	Business Process Management
BPMN	Business Process Modeling Notation
SLR	Systematic Literature Review
RQ	Research Question
fMRI	functional Magnetic Resonance Imaging
EEG	Electroencephalography
MEG	Magnetoencephalography
EMG	Electromyography
CLT	Cognitive Load Theory
TAM	Technology Acceptance Model
U	perceived usefulness
EU	perceived ease of use
CTML	Cognitive Theory of Multimedia Learning
CFT	Cognitive Fit Theory
DCT	Dual Coding Theory
CogniDia	Cognitive Framework of Understanding and Task Performance with Diagrams
SCL	Skin Conductance Level
SCR	Skin Conductance Response
EPC	Event-driven Process Chains
YAWL	Yet Another Workflow Language
UML AD	Unified Modeling Language Activity Diagrams
PMCF	Process Model Comprehension Framework

## Appendix A

**Table A1:** Relevant studies S1–S17.

Study #	Title	Reference	Author
S1	Cognitive insights into business process model comprehension: Preliminary results for experienced and inexperienced individuals	[13]	Michael Zimoch, Rüdiger Pryss, Thomas Probst, Winfried Schlee, Manfred Reichert
S2	Eye tracking experiments on process model comprehension: lessons learned	[41]	Michael Zimoch, Rüdiger Pryss, Johannes Schobel, Manfred Reichert
S3	Using insights from cognitive neuroscience to investigate the effects of event-driven process chains on process model comprehension	[42]	Michael Zimoch, Tim Mohring, Rüdoger Pryss, Thomas Probst, Winfried Schlee, Manfred Reichert
S4	Utilizing the capabilities offered by eye-tracking to foster novices’ comprehension of business process models	[43]	Michael Zimoch, Rüdiger Pryss, Georg Layher, Heiko Neumann, Thomas Probst, Winfried Schlee, Manfred Reichert
S5	The repercussions of business process modeling notations on mental load and mental effort	[44]	Michael Zimoch, Rüdiger Pryss, Thomas Probst, Winfried Schlee, Manfred Reichert
S6	Comprehension of business process models: Insight into cognitive strategies via eye tracking	[45]	Miles Tallon, Michael Winter, Rüdiger Pryss, Katrin Rakoczy, Manfred Reichert, Mark W. Greenlee, Ulrich Frick
S7	Learning to read by learning to write: Evaluation of a serious game to foster business process model comprehension	[46]	Michael Winter, Rüdiger Pryss, Thomas Probst, Manfred Reichert
S8	Towards the applicability of measuring the electrodermal activity in the context of process model comprehension: Feasibility study	[47]	Michael Winter, Rüdiger Pryss, Matthias Fink, Manfred Reichert
S9	Measuring the cognitive complexity in the comprehension of modular process models	[48]	Michael Winter, Rüdiger Pryss, Thomas Probst, Julia Bass, Manfred Reichert
S10	Applying eye movement modeling examples to guide novices’ attention in the comprehension of process models	[49]	Michael Winter, Rüdiger Pryss, Thomas Probst, Manfred Reichert
S11	How healthcare professionals comprehend process models-an empirical eye tracking analysis	[50]	Michael Winter, Cynthia Bredemeyer, Manfred Reichert, Heiko Neumann, Thomas Probst, Rüdiger Pryss
S12	Defining gaze patterns for process model literacy–Exploring visual routines in process models with diverse mappings	[51]	Michael Winter, Heiko Neumann, Rüdiger Pryss, Thomas Probst, Manfred Reichert
S13	Towards measuring and quantifying the comprehensibility of process models: the process model comprehension framework	[52]	Michael Winter, Rüdiger Pryss, Matthias Fink, Manfred Reichert
S14	An Empirical Exploration of Working Memory, Selective Attention and Reasoning During the Comprehension of Process Models	[53]	Michael Winter, Rüdiger Pryss
S15	The effects of modular process models on gaze patterns—A follow-up investigation about modularization in process model literacy	[54]	Michael Winter, Rüdiger Pryss
S16	Exploring Comprehension Strategies of Modular Process Models: A Combined Eye-Tracking and Concurrent Think-Aloud Study	[55]	Julia Baß, Michael Winter, Rüdiger Pryss, Manfred Reichert
S17	A study on the effects of routing symbol design on process model comprehension	[56]	Kathrin Figl, Jan Recker, Jan Mendling

**Table A2:** Relevant studies S18–S38.

Study #	Title	Reference	Author
S18	A study into the factors that influence the understandability of business process models	[57]	Hajo A. Reijers, Jan Mendling
S19	Adopting the cognitive complexity measure for business process models	[58]	Volker Gruhn, Ralf Laue
S20	An integrative framework of the factors affecting process model understanding: a learning perspective	[59]	Hajo A. Reijers, Jan Recker, Sander van de Wouw
S21	Analysing the cognitive effectiveness of the BPMN 2.0 visual notation	[60]	Nicolas Genon, Patrick Heymans, Daniel Amyot
S22	Animation as a dynamic visualization technique for improving process model comprehension	[61]	Banu Aysolmaz, Hajo A. Reijers
S23	Assessing the impact of hierarchy on model understandability—a cognitive perspective	[62]	Stefan Zugal, Jakob Pinggera, Barbara Weber, Jan Mendling, Hajo A. Reijers
S24	Business process and rule integration approaches—An empirical analysis of model understanding	[63]	Wei Wang, Tianwa Chen, Marta Indulska, Shazia Sadiq, Barbara Weber
S25	Cognitive diagram understanding and task performance in systems analysis and design	[19]	Monika Malinova Mandelburger, Jan Mendling
S26	Cognitive style and business process model understanding	[64]	Oktay Turetken, Irene Vanderfeesten, Jan Claes
S27	Don’t overthink it: The paradoxical nature of expertise for the detection of errors in conceptual business process models	[65]	Karl-David Boutin, Christopher Davis, Alan Hevner, Pierre-Majorique Léger, Elise Labonte-LeMoyne
S28	Empirical investigation of the usefulness of gateway constructs in process models	[66]	Jan Recker
S29	Enhancing understandability of process models through cultural-dependent color adjustments	[67]	Tyge-F. Kummer, Jan Recker, Jan Mendling
S30	Eye-tracking the factors of process model comprehension tasks	[68]	Razvan Petrusel, Jan Mendling
S31	Factors of process model comprehension—Findings from a series of experiments	[69]	Jan Mendling, Mark Strembeck, Jan Recker
S32	Findings from an experiment on flow direction of business process models	[70]	Kathrin Figl, Mark Strembeck
S33	How do humans inspect BPMN models: an exploratory study	[71]	Cornelia Haisjackl, Pnina Soffer, Shao Yi Lim, Barbara Weber
S34	How does it look? Exploring meaningful layout features of process models	[72]	Vered Bernstein, Pnina Soffer
S35	How visual cognition influences process model comprehension	[73]	Razvan Petrusel, Jan Mendling, Hajo A. Reijers
S36	Influence factors for local comprehensibility of process models	[74]	Kathrin Figl, Ralf Laue
S37	Influence of diagram layout and scrolling on understandability of BPMN processes: an eye tracking experiment with BPMN diagrams	[75]	Daniel Lübke, Maike Ahrens, Kurt Schneider
S38	Narration as a technique to improve process model comprehension: Tell me what I cannot see	[76]	Banu Aysolmaz, Farida Nur Cayhani, Hajo A. Reijers

**Table A3:** Relevant studies S39–S47.

Study #	Title	Reference	Author
S39	On a quest for good process models: the cross-connectivity metric	[77]	Irene Vanderfeesten, Hajo A. Reijers, Jan Mendling, Wil M.P. van der Aalst, Jorge Cardoso
S40	On the cognitive effects of abstraction and fragmentation in modularized process models	[78]	Clemens Schreiber, Amine Abbad-Andaloussi, Barbara Weber
S41	Process model comprehension: the effects of cognitive abilities, learning style, and strategy	[29]	Jan Recker, Hajo A. Reijers, Sander G. van de Wouw
S42	Reducing the cognitive complexity of business process models	[79]	Volker Gruhn, Ralf Laue
S43	Syntax highlighting in business process models	[80]	Hajo A. Reijers, Thomas Freytag, Jan Mendling, Andreas Eckleder
S44	The effects of content presentation format and user characteristics on novice developers’ understanding of process models	[81]	Jan Recker, Alexander Dreiling
S45	The influence of notational deficiencies on process model comprehension	[82]	Kathrin Figl, Jan Mendling, Mark Strembeck
S46	The influence of using collapsed sub-processes and groups on the understandability of business process models	[83]	Oktay Turetken, Ahmet Dikici, Irene Vanderfeesten, Tessa Rompen, Onur Demirors
S47	Using eye tracking to expose cognitive processes in understanding conceptual models	[84]	Palash Bera, Pnina Soffer, Jeffrey Parsons

**Table A4:** Content of the relevant studies S1–S13.

Study	Content
S1	S1 presents preliminary findings from an eye-tracking experiment aiming to understand how individuals with varying levels of expertise comprehend business process models. By integrating concepts from cognitive neuroscience and psychology, S1 investigates how process model complexity and personal experience influence comprehension.
S2	S2 presents lessons learned from a series of eye-tracking experiments conducted to understand how different process modeling languages (BPMN, eGantt, EPC, Petri Nets) affect process model comprehension.
S3	S3 investigates the effects of EPCs on process model comprehension using insights from cognitive neuroscience. Through an eye-tracking experiment, it examines how expertise in process modeling influences the ability to comprehend EPC models of varying complexity.
S4	S4 presents a study that investigates how eye tracking can be used to improve the comprehension of business process models, particularly for novices. Study S4 compares the eye movements of both novices and experts as they read and comprehend BPMN 2.0 process models.
S5	S5 investigates the cognitive load (i.e., mental load and mental effort) experienced by individuals when comprehending business process models expressed in various notations. Study S5 involves participants (novices, intermediates, and experts) who assessed process models in eight different notations (e.g., BPMN 2.0, EPCs, Petri Nets, and UML Activity Diagrams).
S6	S6 investigates how individuals comprehend business process models by utilizing eye-tracking data to explore cognitive strategies. Two experiments were conducted: one with high school students and another with visual literacy experts and novices. Study S6 aims to identify differences in process model comprehension based on complexity, model design, and visual literacy.
S7	S7 explores the use of a serious game called Tales of a Knightly Process to enhance the comprehension of business process models. Study S7 involves two experiments with university students to measure the immediate and follow-up impacts of the game on their ability to comprehend BPMN 2.0 models.
S8	S8 explores the potential of using electrodermal activity to understand cognitive load during business process model comprehension. Through a feasibility study, S8 examines how individuals’ physiological responses, measured via electrodermal activity, correlate with the complexity of process models.
S9	S9 explores the effects of modularization on the comprehension of business process models. Study S9 investigates three types of modularization (horizontal, vertical, and orthogonal) and their impact on cognitive load, comprehension performance, and acceptability.
S10	S10 presents research on improving the comprehension of process models by using Eye Movement Modeling Examples (EMMEs). EMMEs leverage the eye-tracking data of experts to guide novices in reading and comprehending process models, particularly those using BPMN 2.0 notation. Study S10 compares the performance of novices with and without EMME support.
S11	S11 investigates how healthcare professionals comprehend process models, specifically those using BPMN 2.0 notation. Using eye-tracking technology, S11 evaluates the comprehension process by analyzing participants’ eye movements, cognitive load, and performance on comprehension questions.
S12	S12 investigates how visual routines and gaze patterns contribute to the comprehension of business process models. Using eye-tracking data, S12 analyzes how participants comprehend process models with different layouts (straight, upward, and downward mappings) and complexity levels (easy, medium, hard).
S13	S13 introduces the PMCF, which aims to measure and quantify how process models are comprehended. By integrating perspectives from process modelers and readers, the PMCF identifies pitfalls in the communication of process information that affect model comprehension.

**Table A5:** Content of the relevant studies S14–S24.

Study	Content
S14	S14 investigates the impact of three cognitive abilities (working memory, selective attention, and reasoning) on the comprehension of process models.
S15	S15 explores how different modularization approaches (vertical, horizontal, and orthogonal) impact the comprehension of business process models, focusing on gaze patterns and cognitive load.
S16	S16 investigates how individuals comprehend modular process models using a combination of eye-tracking and concurrent think-aloud methods. Study S16 examines three types of process model modularization: flattened process models, models with grouped elements, and models with subprocesses.
S17	S17 evaluates the impact of four visual design principles (perceptual discriminability, pop-out effects, semantic transparency, and aesthetics) on humans’ comprehension accuracy, efficiency, and perceived task difficulty.
S18	S18 investigates both personal and model-related factors that affect the comprehension of business process models. Study S18 uses a survey conducted with students and professionals to explore how characteristics such as theoretical knowledge, practical experience, and model complexity influence the comprehension of process models.
S19	S19 proposes a method to measure the cognitive complexity of (business) process models by adapting the cognitive weight metric originally designed for software. Study S19 discusses how process models, often visualized using languages like BPMN or YAWL, can be assessed for complexity by assigning cognitive weights to various control structures such as sequences, XOR-splits, and OR-splits.
S20	S20 proposes a framework to understand how humans comprehend business process models. It divides the comprehension process into three stages: Presage, Process, and Product. The framework incorporates user characteristics (e.g., personality, motivation, skills) in the Presage stage, cognitive processes and strategies in the Process stage, and learning outcomes (e.g., retention and transfer) in the Product stage. S20 emphasizes that the comprehension of process models is an interactive learning process influenced by user-specific attributes and proposes strategies to enhance model comprehension based on these findings.
S21	S21 evaluates the cognitive efficiency of the BPMN 2.0 visual notation using the Physics of Notations framework. Study S21 identifies several issues with the current BPMN design, such as poor perceptual discriminability and semiotic clarity, which can hinder effective model comprehension. S21 proposes improvements, including refining symbol clarity, increasing visual expressiveness, and enhancing modularization techniques to better support different user expertise levels and task types.
S22	S22 explores the use of animation to enhance the comprehension of process models. Building on cognitive load theory and the cognitive theory of multimedia learning, S22 examines how animation can reduce cognitive overload and improve comprehension, particularly for humans with varying levels of expertise.
S23	S23 explores the effects of hierarchical structuring (e.g., modularity and decomposition) on the comprehensibility of conceptual models. Study S23 identifies two key cognitive factors influencing comprehension: abstraction, which reduces cognitive load by summarizing information, and the split-attention effect, where attention is divided between sub-models, increasing cognitive effort.
S24	S24 examines the impact of integrating business rules directly into business process models, particularly using a method called rule linking, where rules are visually connected to process elements. Study S24 uses eye tracking to compare the comprehension efficiency between integrated and separated rule approaches.

**Table A6:** Content of the relevant studies S25–S38.

Study	Content
S25	S25 develops a theoretical framework called CogniDia to understand cognitive processing when using diagrams in systems analysis and design. S25 integrates cognitive theories such as dual coding, cognitive fit, and multimedia learning to explain how humans comprehend and utilize diagrams for systems analysis and design tasks.
S26	S26 examines the impact of cognitive styles and theoretical knowledge on the comprehension of business process models. It uses the Cognitive Style Index to classify participants into intuitive, analytical, and adaptive cognitive styles.
S27	S27 investigates how expertise influences the detection of errors in business process models. Study S27 examines the differences between experts and novices when identifying semantic and syntactic errors.
S28	S28 examines how gateway constructs, like those used in BPMN to indicate parallel splits and simple merges, influence process model comprehension.
S29	S29 investigates how culturally tailored color schemes in process models affect comprehension. S29 compares participants from Confucian (Chinese) and Germanic (German and Austrian) cultures, finding that culturally preferred color schemes improve comprehension efficiency for Confucian participants but have mixed effects on Germanic participants.
S30	S30 explores how process model comprehension can be influenced by identifying relevant regions within models, areas that contain essential information for answering comprehension questions.
S31	S31 explores how various factors influence the comprehension of business process models. Study S31 uses a series of experiments to investigate both model-related factors, such as model complexity and the type of information presented, and personal factors, including theoretical knowledge and modeling experience.
S32	S32 investigates how different flow directions (left-to-right, right-to-left, top-to-bottom, and bottom-to-top) affect the comprehension of business process models. S32 involved a controlled experiment to test whether specific flow directions improve comprehension accuracy and efficiency.
S33	S33 investigates how individuals identify and classify quality issues in BPMN process models. Study S33 employs a think-aloud method to analyze the cognitive strategies used by participants when inspecting models for syntactic, semantic, and pragmatic errors.
S34	S34 investigates the impact of layout features on the comprehensibility of process models. Study S34 highlights that while certain layout aspects, such as edge crossings and alignment, are commonly acknowledged, a systematic understanding of their effects on cognitive load is lacking.
S35	S35 explores the role of visual cognition in the comprehension of process models. Using an eye-tracking experiment, S35 investigates how model-related factors (such as complexity) and person-related factors (such as expertise) are mediated by visual cognition variables.
S36	S36 investigates the factors affecting the local comprehensibility of process models. S36 focuses on deductive reasoning tasks and identifies how the presence of different control-flow patterns (e.g., sequences, loops, parallel execution) and the interaction between model elements impact cognitive difficulty.
S37	S37 investigates how different diagram layouts and the necessity of scrolling affect the comprehension of BPMN models. Through an eye-tracking experiment with professional software developers, S37 compares horizontal and vertical layouts, as well as variations that require or avoid scrolling.
S38	S38 investigates the use of narration to enhance the comprehension of process models. By leveraging the dual coding theory and cognitive theory of multimedia learning, S38 explores how auditory and visual information channels can be combined to alleviate cognitive load.

**Table A7:** Content of the relevant studies S39–S47.

Study	Content
S39	S39 introduces the cross-connectivity metric as a means to evaluate the quality and comprehensibility of process models. the metric assesses the strength of connections between model elements, with the hypothesis that higher connectivity leads to better comprehension and fewer errors.
S40	S40 investigates the impact of modularization on process model comprehension, focusing on the cognitive effects of abstraction and fragmentation. Through an empirical eye-tracking study, S40 analyzes how humans engage with modularized models when performing local tasks (focused on a single module) versus global tasks (spanning multiple modules).
S41	S41 investigates how various cognitive abilities, such as abstraction and selection skills, influence the comprehension of process models.
S42	S42 discusses methods for enhancing the comprehensibility of business process models by minimizing cognitive load. Study S42 proposes patterns that can be applied to simplify models, such as replacing complex gateways (e.g., or-gateways) with simpler constructs (e.g., xor-gateways) when possible, and eliminating redundant elements or unnecessary sequences.
S43	S43 explores the application of syntax highlighting techniques to improve the comprehension of business process models, particularly within the context of workflow nets. Study S43 introduces a method to color code critical elements such as operator transitions (e.g., xor, and splits/joins) to enhance visibility and reduce cognitive load.
S44	S44 explores how different presentation formats (e.g., EPC vs. BPMN) and user characteristics (such as experience and language proficiency) influence the comprehension of process models by novice developers.
S45	S45 investigates how notational deficiencies in process modeling languages, specifically concerning perceptual discriminability and semiotic clarity, impact cognitive load and model comprehension.
S46	S46 investigates how different forms of vertical modularization in BPMN (fully flattened, grouped elements, and collapsed sub-processes) affect the comprehensibility of process models.
S47	S47 utilizes eye-tracking technology to investigate the cognitive processes involved in comprehending conceptual modeling scripts, such as BPMN and EPC. Study S47 presents two experimental studies: the first compares scripts generated using different modeling grammars, showing how variations in attention to specific parts of the models affect task performance. The second study combines eye tracking with verbal protocol analysis to explore how visual association between elements of a model indicates cognitive integration during problem-solving tasks.
